# Relapsed/Refractory Diffuse Large B-Cell Lymphoma: Is There Still a Role for Autologous Stem Cell Transplantation in the CAR T-Cell Era?

**DOI:** 10.3390/cancers16111987

**Published:** 2024-05-23

**Authors:** Tim Strüßmann, Reinhard Marks, Ralph Wäsch

**Affiliations:** Department of Medicine I, Faculty of Medicine, Medical Center–University of Freiburg, University of Freiburg, 79106 Freiburg, Germany; reinhard.marks@uniklinik-freiburg.de (R.M.); ralph.waesch@uniklinik-freiburg.de (R.W.)

**Keywords:** CAR T-cell therapy, DLBCL, autologous stem cell transplantation

## Abstract

**Simple Summary:**

Chimeric antigen receptor (CAR) T-cell therapies dramatically changed the treatment strategies for relapsed/refractory diffuse large B-cell lymphoma and displaced autologous stem cell transplantation as the standard of care for patients with refractory disease or early relapse. However, for patients responding to salvage therapy, autologous stem cell transplantation remains a valid therapy approach.

**Abstract:**

Recently, CD19-directed chimeric antigen receptor (CAR) T-cell therapies have revolutionized treatment strategies for diffuse large B-cell lymphoma (DLBCL). CAR T-cell therapy is increasingly used as a second-line therapy for patients with DLBCL with early relapse or refractoriness to initial chemoimmunotherapy and displaced high-dose chemotherapy, followed by autologous stem cell transplantation (ASCT) as the standard of care for these patients. However, patients with late relapse or chemosensitive disease still benefit from autologous stem cell transplantation. We will review practice-changing studies in early relapse (ZUMA-7 and TRANSFORM) under consideration of the negative BELINDA trial, with a focus on register data, comparing CAR T-cell therapy and ASCT for patients responding to salvage therapy.

## 1. Introduction

Diffuse large B-cell lymphoma (DLBCL) is the most prevalent subtype of non-Hodgkin lymphoma and is characterized by distinct molecular profiles and aggressive behavior. The prognosis of patients with DLBCL is influenced by various clinical and molecular factors, including cell-of-origin subtype, protein overexpression, and chromosomal rearrangements [1,2,3]. Despite significant advancements in first-line treatment, approximately 10% of patients do not respond to therapy, and up to one-third of those in complete remission eventually relapse [4,5,6].

For the past 25 years, high-dose chemotherapy followed by autologous stem cell transplantation (ASCT) has been the standard-of-care (SOC) second-line therapy for patients with DLBCL responding to chemotherapy [7,8]. However, patients with prior rituximab exposure, primary refractory disease, or early relapse within 12 months after completing therapy have an extremely poor prognosis [9,10,11]. The role of ASCT in the rituximab era may diminish, particularly in patients with refractory disease or early relapse. Chemotherapy refractoriness was not overcome by ASCT. With the advent of chimeric antigen receptor T-cell therapies, it is now possible to achieve long-term disease-free survival in patients with relapsed or refractory DLBCL. Axicabtagene ciloleucel (axi-cel), tisagenlecleucel (tisa-cel), and lisocabtagene maraleucel (liso-cel) have been approved for patients with DLBCL with relapsed or refractory disease after two or more lines of therapy, achieving durable remissions in approximately 40% of patients [12,13,14]. Recently, three phase-3 trials have challenged the standard of care in the DLBCL second-line treatment [15,16,17]. Although CAR T-cell therapies have superseded ASCT as the standard of care for patients with refractory or early-relapse DLBCL, high-dose chemotherapy followed by autologous stem cell transplantation remains an option for patients with chemosensitive early or late relapse.

## 2. Second-Line Immunochemotherapy and High-Dose Chemotherapy Followed by Autologous Stem Cell Support: A Quarter-Century-Long Standard of Care for Patients with r/r DLBCL

In recent decades, the standard of care for relapsed and refractory DLBCL was to administer second-line (immuno-)chemotherapy and attain high-dose chemotherapy, followed by autologous stem cell support.

Published in 1995, the randomized Parma trial clearly demonstrated the superior effectiveness of ASCT in comparison to conventional chemotherapy with DHAP; the response rate was 84% after ASCT and 44% after conventional chemotherapy without transplantation. Moreover, the 5-year event-free survival rate was 46% in the transplantation group compared to 12% in the chemotherapy only group [8]. This trial laid the foundation for ASCT as the standard of care, even though it was carried out in the pre-rituximab era.

However, there were several obstacles: First of all, patients had to achieve a remission before proceeding to transplant; performing ASCT in chemo-insensitive patients was shown to be unsuccessful [7]. The most common salvage regimens are platinum-based R-DHAP (rituximab, dexamethasone, cytarabine, cisplatin), R-ICE (rituximab, ifosfamide, carboplatin, etoposide), or R-GDP (rituximab, gemcitabine, dexamethasone, cisplatin). All these regimens showed similar efficacy in achieving a response as second-line salvage therapy. R-ICE and R-DHAP led to response rates of 63 and 64%, respectively, in the CORAL trial [9]. R-GDP was not tested head-to-head with R-ICE but showed similar response rates to R-DHAP in the LY.12 trial [10]. However, patients with early relapse (<12 months from diagnosis and prior rituximab exposure) in the CORAL trial or refractory disease had a dismal outcome. The multicenter retrospective SCHOLAR-1 study confirmed the dismal prognosis of refractory DLBCL. Refractoriness was defined as progressive disease or stable disease as best response at any time point during chemotherapy (>four cycles of first-line therapy or two cycles of later-line therapy), and relapse at 12 months from ASCT and resulted in a 26% overall response rate, with a 7% complete remission rate and median overall survival (OS) of six-month [11].

Another limitation of the ASCT approach is the need for transplant eligibility, as high-dose chemotherapy is associated with relevant toxicities [8]. In the context of aging societies and the median age of 64 years at DLBCL diagnosis, one could expect that probably one half of patients with r/r DLBCL may not be medically fit for ASCT [18,19].

Taking these points into consideration, it is obvious that overcoming chemotherapy resistance is a medical need. In recent years, several novel therapies have demonstrated efficacy in r/r DLCBL and are in the starting blocks to challenge the standard of care. Most promising approaches are immunotherapy-based, e.g., CAR T-cell therapy or bispecific antibody therapy (CD19/CD3 bispecific T-cell engager (BiTE)) [12,13,14,20,21,22].

## 3. CAR T-Cell Therapy and the Practice-Changing Studies in Early DLBCL Relapse (ZUMA-7 and TRANSFORM) and Potential Reasons for the Different Outcome of BELINDA

### 3.1. CAR T-Cell Therapy: Efficacy in r/r Large B-Cell Lymphoma and Side-Effects

CAR T-cell therapy is a groundbreaking immunotherapy. This innovative approach involves genetically modified T-cells that are programmed to target the B-cell marker CD19. The three currently approved products, axicabtagene ciloleucel, tisagenlecleucel, and lisocabtagene maraleucel, have distinct design features, such as differences, e.g., in the co-stimulatory domain or mechanism of viral transduction [12,13,14].

Results from the pivotal trials, ZUMA-1, JULIET, and TRANSCEND led to the approval of these products in patients with relapsed/refractory DLBCL. All three CAR T-cell products have demonstrated the ability to induce durable remissions in approximately 30–40% of treated patients, including those who had not been in a durable remission with a prior ASCT, whereas the longest median follow-up differs (TRANSCEND: 20 m; JULIET: 40.2 m; and ZUMA-1 with 63.1 m) [12,13,14]. These products have been approved by the US Food and Drug Administration (FDA) and European Medical Association (EMA) for patients with r/r DLBCL and other large B-cell lymphomas (LBCL) after at least two lines of therapy. Product-specific features, outcomes, and toxicities are summarized in Table 1.

Registry data have confirmed CAR T-cell effectiveness [23,24,25]. A real-world analysis of the retrospective French DESCAR-T registry, including 809 patients, indicated the inferior efficacy of tisa-cel compared to axi-cel in the third or later line of therapy (1-year progression-free survival (PFS) was 46.6% for axi-cel and 33.2% for tisa-cel) (hazard ratio (HR) = 0.61; 95% confidence interval (CI), 0.46–0.79; *p* = 0.0003) [26].

CAR T-cell therapies have a specific side-effect profile. Most important in this context are cytokine release syndrome (CRS) and immune-effector-cell-related neurotoxicity syndrome (ICANS). The frequency of CRS and ICANS varies between the different products. CRS is a supraphysiologic immune response with activation and engagement of T-cells and other immune effector cells. Patients typically have fever and may develop multi-organ failure in higher-grade CRS [27]. CRS severity has been associated with the dose of CAR T-cells and their expansion [28]. The design of the CAR (e.g., 4-1BB vs. CD28 co-stimulatory domain) also may affect the frequency and severity of CRS [29]. Higher-grade CRS can be mitigated by interrupting interleukin-6 signaling with IL-6-receptor antagonist tocilizumab [30]. Across all CAR T-cell products, ICANS is less common than CRS, but highest ICANS rates are described with axi-cel (≥ grade 3: 28% vs. 10–11% for liso-cel and tisa-cel, respectively) [12,13,14,31]. These observations largely depend on the different co-stimulatory domains of the products (axi-cel: CD28; tisa-cel and liso-cel: 4-1BB), whereby CD28 led to more rapid T-cell expansion and effector/memory T-cell differentiation [32], with a higher risk of neurotoxicity. Therapy of ICANS contains supportive care and, if necessary, intensive care, dexamethasone, high-dose methylprednisolone, and/or interleukin-2 blockade, depending on severity [33].

Rare cases of T cell malignancies are emerging in patients who received CD19 directed CAR T-cell therapy. In some of these lymphomas, the malignant cells are CAR-positive [34]. The actual incidence of secondary T-cell lymphomas after CAR T-cell therapy is not well defined, and it is uncertain as to whether these tumors are associated with CAR T-cell products.

### 3.2. ZUMA-7, TRANSFORM, and BELINDA Trials and Approval of CAR T-Cell Products in Early Relapsed/Refractory DLBCL

#### 3.2.1. Challenging the Standard of Care in Early Relapsed and Refractory

Given the proven efficacy of CAR T-cell therapy in relapsed or refractory large B-cell lymphoma, it was hypothesized that CAR T-cell therapy could change the standard of care for patients with early relapse or primary refractory disease. The three FDA-approved CAR T-cell therapies were intended to undergo a randomized comparison with salvage therapy followed by autologous stem cell transplantation in ZUMA-7 (axi-cel), BELINDA (tisa-cel), and TRANSFORM (liso-cel) trials, respectively [15,16,17].

The trials shared substantial similarities, including the enrolment of patients with large B-cell lymphoma from the age of 18 years with early relapse (within 12 months from the end of therapy) or primary refractory disease. The main inclusion criteria were similar, with the exception of an upper age limit of 75 years in TRANSFORM. Notably, in contrast to TRANSFORM and BELINDA, the ZUMA-7 trial did not allow a crossover; however, the patients were able to receive CAR T-cell therapy in the study. Moreover, bridging therapy (except for steroids for disease control) was not allowed in ZUMA-7, whereas patients could receive bridging therapy with the same regimens as the standard-of-care arm in BELINDA and TRANSFORM. Of note, in TRANSFORM, only one cycle of chemoimmunotherapy was allowed, while BELINDA allowed for more cycles and a switch of regimes.

Event-free survival (EFS) was the primary endpoint of all three trials, but the definition of EFS differed; stable disease at different time points (ZUMA-7: day 150; BELINDA: week 12; TRANSFORM: week 9) was included, in addition to death and progressive disease. Notably, the initiation of a new therapy was considered an event in ZUMA-7 and TRANSFORM but not in BELINDA. The key trial features are presented in Table 2.

#### 3.2.2. Patients and Outcomes

In the ZUMA-7 trial, 180 patients were randomly assigned to receive axi-cel and 179 to receive the standard of care. The median age was 59 years (range 21–81), and 74% of patients had refractory disease. As many as 19% of patients had high-grade B-cell lymphoma with MYC and BCL2 or BCL6-Rearrangement. As many as 54% of patients had elevated lactate dehydrogenase levels. Regarding CAR T-cell product delivery, 94% of the patients in the axi-cel arm received the product. The median time from randomization to infusion was 29 days. A total of 36% (65 patients) required bridging therapy with steroids. In the standard-of-care arm, 94% of patients received platinum-based salvage therapy, but notably, only 36% of patients underwent autologous stem cell transplantation. The median EFS was significantly longer in the axi-cel group (8.3 vs. 2.0 months; hazard ratio for event or death: 0.40; 95% CI: 0.31 to 0.51; *p* < 0.001) [15]. In the prespecified overall survival analysis at 5 years, the median overall survival was not reached in the axi-cel group, while it was 31.1 months in the standard-of-care group. The estimated 4-year overall survival was superior in the axi-cel group, with 54.6% compared to 46.0% in the standard-care group. The hazard ratio for death was 0.73 (95% CI: 0.54 to 0.98; *p* = 0.03) [35]. Notably, in addition to the reported survival benefit, axi-cel showed meaningful improvements in quality of life (QoL) compared to SOC [36].

In the BELINDA trial, a total of 322 patients were randomized, 162 to the tisa-cel arm and 160 to the standard of care arm. Baseline characteristics were well balanced, with slightly more cases of high-grade B-cell lymphoma with MYC and BCL2 or BCL6 rearrangement in the tisa-cel arm (20% vs. 12%). A total of 66% of patients in both groups had primary refractory disease. LDH levels were not reported. As many as 83% of patients in the tisa-cel arm received bridging chemoimmunotherapy. Notably, at week six, 26% of patients in the tisa-cel arm had progressive disease prior to CAR T-cell infusion compared to 14% in the standard-of-care arm. However, 155 of 162 patients received tisa-cel. In addition, the median time from randomization to tisa-cel infusion was 52 days. More than half of the patients in the SOC arm received at least two salvage regimens, but only 32% of the patients received autologous stem cell transplantation; however, 50% of the patients in the standard-of-care arm crossed over and received tisa-cel. With a median follow-up of 10 months, EFS at 3 months was not significantly different in the two groups (hazard ratio for event or death: 1.07; 95% CI: 0.82–1.40; *p* = 0.61) [16].

The TRANSFORM trial enrolled 184 patients, with 92 patients assigned to the liso-cel group and 92 to the standard of care group. The median ages were 60 and 58 years in the liso-cel and standard of care arms, respectively. Baseline characteristics were well balanced, with more than 70% of patients having primary refractory disease. As many as 63% of patients in the liso-cel arm received one cycle of bridging chemoimmunotherapy, and nearly 98% received the CAR T-cell product. In the standard-of-care arm, only 46% of patients received autologous stem cell transplantation. In the primary analysis, the median follow-up period was 17.5 months, and the median event-free survival (EFS) was not reached for the liso-cel group, while it was 2.4 months for the SOC group. The complete response rate was 74% for the liso-cel group compared to 43% for the SOC group (*p* < 0.0001), and the median progression-free survival (PFS) was not reached for the liso-cel group, while it was 6.2 months for the SOC group (hazard ratio = 0.400; *p* < 0.0001). The reported rates of severe CRS and NEs (CTCAE Grade ≥ 3) were lower for liso-cel than those reported with axi-cel in ZUMA-7 [15,17,37].

Taking ZUMA-7 and TRANSFORM together led to the FDA and EMA approval of axi-cel and liso-cel for large B-cell lymphoma patients with early relapse or primary refractory disease. The difference in the efficacy of tisa-cel compared to axi-cel and liso-cel could, in part, be due to differences in the mode of action between the three products, but there could also be methodological reasons that explain the different results. In the ZUMA-7 trial, the enrolled patients might a have less-aggressive disease since they were not intended to receive bridging therapy, except for steroids. This might have led to a decreased enrollment of patients who needed urgent therapy. Patients who do not need systemic bridging therapy have a better outcome compared to those who do need one [38]. Additionally, the patients on the BELINDA trial might have has a more progressive disease at the point of CAR T-cell infusion as the median time to CAR T-cell infusion was 59 days. Moreover, they might have a more refractory disease as ≥1 lines of bridging therapy were allowed. Another factor is the difference in EFS definitions in all three trials, as stable disease was considered an event at week 9 in the TRANSFORM trial, at week 12 in the BELINDA trial, and at week 21 in the ZUMA-7 trial. Though the efficacy of the CAR T-cell product was earlier assessed in the TRANSFORM and BELINDA trial, the time given to respond was probably long enough in the ZUMA-7 trial. In light of the extended time period from randomization to CAR T-cell infusion in the BELINDA trial, it might be possible that the EFS was assessed before tisa-cel could act to its full potential, as the median time to response was 2 months in the JULIET trial [13]. This also highlights that EFS as the primary endpoint remains controversial; the definitions vary among the three trials, and it is not a validated surrogate for overall survival instead of progression-free survival [39]. However, with a longer follow-up of 4 years, an overall survival benefit was reported with a hazard ratio (HR) of 0.73 (95% CI: 0.54 to 0.98; *p* = 0.03) for axi-cel. And with a follow-up of 17.5 months, liso-cel showed a superior progression-free survival (hazard ratio (HR) = 0.400; *p* < 0.0001), which might transform into a superior OS with longer follow-up.

## 4. CAR T-Cell Therapy vs. Autologous Stem-Cell Transplant in Patients Responding to Salvage Therapy

### Autologous Transplant vs. CAR T-Cell Therapy for Relapsed DLBCL in Partial Remission

In light of the move of CAR T-cell therapy to the second line for patients with early relapse or primary refractory disease, Shadman et al. [40] performed a retrospective Center for International Blood and Marrow Transplant (CIBMTR) database analysis and compared the outcomes of autologous stem-cell transplant (ASCT) and CAR T-cell therapy for patients in partial remission:

Patients with DLBCL received either an ASCT (2013–2019) or CAR T-cell therapy with axi-cel (2018–2019), while a PR was demonstrated via computer tomography (CT) or positron emission tomography (PET). Patients with an available negative PET (Deauville 1–3) or patients with a prior ASCT in the CAR T-cell cohort were excluded from the analysis. A total of 411 patients were identified, of whom 266 received an ASCT and 145 a CAR T-cell therapy. Regarding baseline characteristics, there was no difference in age, performance status, and the proportion of patients who had a pretreatment PET scan. ASCT patients had received fewer median lines of prior therapy (2 vs. 3; *p* < 0.001), and fewer patients in the ASCT group had the largest pretreatment residual node, measuring >5 cm (29% vs. 41%; *p* = 0.05), which might indicate that more patients had a more active lymphoma in the CAR T-cell group. A total of 14 patients received CAR T-cell therapy after ASCT.

In the univariate analysis, the 2-year progression-free survival (52% vs. 42%; *p* = 0.1) and the rate of 100-day non-relapse mortality (4% vs. 2%; *p* = 0.3) were not significantly different. ASCT was associated with a lower rate of relapse and progression (40% vs. 53%; *p* = 0.008) and a superior overall survival (69% vs. 47%; *p* = 0.004) at two years. In the subgroup analysis of patients with ≤2 lines of prior therapy, there was no difference in PFS, cumulative incidence of relapse/progression, or OS. When focusing on patients with early treatment failure (primary refractory disease or relapse within 12 months of diagnosis), ASCT and CAR T-cell cohorts had no significant difference in 2-year PFS (53% vs. 40%; *p* = 0.05), but the ASCT group had lower incidence of relapse/progression (38% vs. 56%; *p* = 0.006) and superior OS (66% vs. 40%, *p* = 0.003) at 2 years compared to the CAR T-cell therapy group. In the multivariate regression analysis, ASCT treatment was associated with a lower risk of relapse/progression (hazard ratio = 1.49; *p* = 0.01) and a superior overall survival (hazard ratio = 1.63; *p* = 0.008) [40].

In line with these findings, Shadman and colleagues retrospectively investigated the outcomes of patients with LBCL achieving a complete remission prior with ASCT (2015–2021; *n* = 281) vs. CAR T-cell therapy (2018–2021; *n* = 79). Results were only available on an abstract basis; patients with CAR T-cell therapy were more likely to have more lines of therapy (3 vs. 2, *p* < 0.01), early treatment failure (72% vs. 58%; *p* = 0.02), and elevated LDH before treatment (37 vs. 31%, *p* = 0.04). In the univariate analysis, treatment with CAR T-cell therapy was associated with a higher rate of relapse at 2 years (48% vs. 27.8%; *p* < 0.001), a lower rate of 2 years PFS (47.8% vs. 66.2%; *p* < 0.001), and lower 2 year OS (65.6 vs. 78.9%; *p* = 0.037). When focusing on patients with early treatment failure, the same was true for the 2-year relapse rate and PFS, while there was no difference in OS. In the multivariate regression analysis, treatment with CAR T-cells compared to ASCT showed a higher risk of relapse (hazard ratio: 2.18; *p* <0.0001) and an inferior PFS (hazard ratio: 1.83; *p* = 0.0011), while there was no difference for OS (hazard ratio: 1.44; *p* = 0.12) [41] Table 3.

## 5. Discussion

When considering the question of whether autologous stem cell transplantation still plays a role in the CAR T-cell era, one must say yes, but various scenarios can be considered here, and they are outlined below.

For patients with refractory disease or early relapse without response to salvage therapy, the situation seems clear: these patients have a very poor prognosis with autologous stem cell transplantation alone, with a median overall survival of 6.3 month [11]. In this case, the patients should be treated with CAR T-cells on the basis of the ZUMA-7 and TRANSFORM studies, which showed a clear survival benefit, including overall survival for ZUMA-7 [35,37].

For patients with a late first relapse (>12 month after primary therapy), no randomized trials have compared ASCT with CAR T-cell therapy, but ASCT has been associated with long-term survival in at least 50% of patients with first relapse and, furthermore, has been associated with better survival than salvage chemotherapy alone [8]. Recently, Hilton and colleagues showed that relapse time (late relapse > 2 years post diagnosis vs. primary refractory or early relapse) is associated with distinct evolutionary dynamics. Their results strongly suggest that late DLBCL relapses commonly occur from a persistent common precursor cell (CPC). This results in a genetically distinct and chemotherapy-naive disease and may, in part, explain the favorable outcome with ASCT [42]. To determine whether a specific time-to-relapse interval can be defined that reliably differentiates patients whose disease represents true r/r DLBCL from new DLBCL arising from a CPC population, larger cohorts than those evaluated in this study will be required. In future, molecular characterization of the lymphoma at relapse could be a step towards a precision medicine approach, as mutations and specific pathway perturbations may differ from those observed at diagnosis.

Whenever medically fit, patients with DLBCL with a second or later relapse should undergo in-label therapy with axi-cel, tisa-cel, or liso-cel [13,14,24]. However, based on a real-world analysis of the French DESCAR-T registry, tisa-cel, when compared to axi-cel, might be less effective after two or more lines of therapy [26]. In addition, a systematic review and meta-analysis by Jacobson and colleagues evaluated the real-world evidence for commercial CAR T-cell products. Their analysis indicates that axi-cel was associated with improved OS and PFS and an increased risk of ICANS grade ≥ 3 compared to tisa-cel [43]. Notably, the phase-2 PILOT trial investigated liso-cel in transplant-ineligible patients; the high ORR and durability of responses in patients who achieved a CR in this trial supported the FDA’s decision to expand their approval of the second-line use of liso-cel to include the transplant-ineligible patient subgroup [44]. A retrospective analysis found real-world evidence of the favorable efficacy of CAR T-cell therapy in elderly patients with relapsed/refractory LBCL. Durable remission rates were similar to those reported in the clinical trial literature [45].

The question remains as to whether patients with early relapse or a primary refractory course and at least a partial remission to salvage therapy should not undergo autologous transplantation after all; in fact, the efficacy of ASCT has been confirmed in a 2021 CIBMTR analysis showing a 41% 5-year PFS in patients with PET-positive PR receiving an ASCT irrespective of the time of initial relapse or primary refractory disease status [46]. This is in line with previous studies reporting a 3-year PFS and OS of 49% and 54%, respectively, for Deauville 4 patients after salvage therapy [47]. Shadman and colleagues, for the subgroup of patients with early treatment failure, reported no significant difference in 2-year PFS but a lower relapse/progression rate and superior OS with ASCT compared to CAR T-cell treatment [40]. However, it must be taken into account that this is a retrospective analysis with the expected biases; in particular, the cohorts were not entirely balanced, and PR definition may differ, especially in the non-clinical trial setting. This may have led to the inclusion of a range of different patients with PR. For patients achieving a CR after salvage therapy, ASCT is associated with a lower relapse rate and an improved PFS compared with CAR T-cell therapy (including patients with early treatment failure). Of note, 13.2% of ASCT patients in this study had a subsequent CAR T-cell therapy, but no patients from the CAR T-cell cohort had a subsequent ASCT [41]. However, in both retrospective studies by Shadman and colleagues, patients treated with CAR T-cells had significantly more lines of prior therapy compared to patients with ASCT. This may have influenced the superior outcomes with ASCT to some extent. Another large retrospective study of patients with r/r DLBCL with R-ICE-based salvage therapy reported no difference in PFS or OS for patients undergoing ASCT in CR, irrespective of time of relapse. This study is currently only available on an abstract basis [48]. There are also reports indicating the efficacy of CAR T-cell therapy in patients with a complete metabolic response; Strati et al. reported thirteen patients with r/r LBCL achieving a complete metabolic response (CMR) prior to CAR T-cell therapy with comparable clinical outcomes and CAR T-cell expansion levels to matched cohorts of patients with active LBCL [49]. In 2019, Bishop et al. reported the outcomes of seven patients with no residual disease prior tisa-cel infusion [50]. Five patients (71%) remained in remission 1 year after treatment. Another 33 patients in complete remission at the point of CAR T-cell therapy were reported by Wudhikarn et al.; eleven patients (33.3%) had a previous ASCT, and two patients (6.1%) had a previous allogeneic transplantation. With a median follow-up of 16 months, 39.4% of patients relapsed and 18.1% died; this resulted in a favorable 1-year EFS and OS of 59.6% and 81.3%, respectively [51]. However, the patient numbers of the reports were relatively small. To our knowledge, there are no prospective trials available in patients with a CMR undergoing CAR T-cell therapy.

Most clinicians will perform bridging therapy before CAR T-cell therapy, which may be necessary for lymphoma control and may have the advantage of reducing the lymphoma burden. This will frequently lead to a situation in clinical practice whereby patients achieve partial or complete remission before the planned cellular therapy. ASCT after achieving a CR is highly effective [41,47] and may be an option as CAR T-cell therapy can be performed after failure of ASCT, whereas it is more uncommon to perform ASCT after CAR T-cell therapy. However, for patients with refractory disease or early relapse, irrespective of their response to bridging treatment, CAR T-cell therapy with axi-cel or liso-cel should be the preferred treatment options due to the clear survival benefit compared to the SOC [35,37]. To clarify the optimal therapeutic approach to patients with remission after bridging/or salvage therapy, the authors advocate exploring ASCT and CAR T-cell therapy in patients with PR/CR in a prospective trial, ideally with molecular studies of the lymphoma prior to cellular therapy and at the time of the potential relapse.

Despite the significant improvement in the second-line treatment of DLBCL with the introduction of CAR T-cell therapy, less than half of all CAR T-cell-treated patients with DLBCL achieve long-term remissions. In addition to baseline parameters such as elevated LDH levels or high metabolic tumor volume, which reflect disease burden, CAR T-cell kinetics, such as CAR T-cell peak levels and subpopulations of circulating CAR T-cells, influence the outcome of CAR T-cell therapy [52,53]. Lymphoma-specific mutations (e.g., TP53) can affect CAR T-cell cytotoxicity, resulting in inferior CAR T-cell therapy efficacy [54]. To optimize CAR T-cell treatment, several clinical trials are ongoing; the phase-3 ZUMA-23 (NCT05605899) evaluates axi-cel vs. SOC in the first line of therapy, which could redefine the standard of care. The phase-1/2 PLATFORM trial (NCT03310619) investigates liso-cel + durvalumab (PD-1-Inhibitor) + Iberdomide (E3 ligase modulator) in r/r DLBCL to overcome lymphoma resistance. There are still unmet clinical needs in the field of CAR T-cell therapy: manufacturing, optimization of bridging therapy, optimization of side-effect management, treatment failure, and management of relapse after CAR T-cell therapy. However, there are ways to overcome these barriers, such as the development of allogeneic CAR T-cell products, CAR T-cell products targeting other antigens (e.g., CD22), and immunomodulatory strategies. CAR T-cell therapies have dramatically changed the treatment of relapsed/refractory DLBCL, and clinical trials are underway to further optimize CAR T-cell therapy.

## 6. Conclusions

CAR T-cell therapy has revolutionized the treatment of relapsed/refractory DLBCL. Particularly in the group of patients with refractory disease or early relapse, significant survival benefits have been demonstrated compared to the previous SOC. Nevertheless, autologous stem cell transplantation remains the standard of care for medically fit patients with late relapse and response to salvage therapy. For patients who respond well to salvage chemotherapy regardless of the time of relapse, ASCT can be discussed, especially for patients in complete remission. However, more prospective data are needed to better guide treatment in this situation.

## Figures and Tables

**Table 1:** Initially approved CAR T-cell therapies in DLBCL.

Product	Axi-Cel	Tisa-Cel	Liso-Cel
Phase-2 trials	ZUMA-1	JULIET	TRANSCEND NHL 001
Co-stimulatory domain	CD28	41BB	41BB
Viral vector	γ-retrovirus	Lentivirus	Lentivirus
Toxicities:			
CRS: any grade ≥grade 3 in %	93	58	42
	13	22	2
ICANS any grade ≥grade 3 in %	64	21	30
	28	12	10
Outcome			
ORR/CR in %	83/58	52/39	73/53
Median PFS (month)	5.9	2.9	6.8
Median OS (month)	25.8	11.1	27.3
Median duration of response (month)	11.1	not reached	23.1
Median follow-up (month)	63.1	40.2	20

**Table 2:** Key trial features of ZUMA-7, BELINDA, and TRANSFORM.

	Axi-Cel	Tisa-Cel	Liso-Cel
Phase-3 trials	ZUMA-7	BELINDA	TRANSFORM
Experimental arm	Axicabtagene ciloleucel	Tisagenlecleucel	Lisocabtagene maraleucel
Primary Endpoint	EFS	EFS	EFS
Event-free survival definition	Time from randomization to: - PD/Death - <PR at day 150 assessment - Start of new lymphoma therapy	Time from randomization to: - PD/Death - <PR at/after week 12	Time from randomization to: - PD/Death - <PR at week 9 - Start of new lymphoma therapy
Bridging therapy prior CAR T-cell therapy	Dexamethasone ≤ 40 mg for ≤4 d	SOC platinum-based therapy	Single cycle of SOC platinum-based therapy
Received bridging therapy (%) Experimental arm	36	83	63
1 line		71	63
≥2 lines		12	
SOC	R-DHAP/R-ICE/R-GDP/R-ESHAP	R-DHAP/R-ICE/R-GDP/R-GemOx	R-DHAP/R-ICE or R-GDP
Cross over	not planned	allowed	allowed
Received intended CAR T-cell therapy (%)	94	96	97.8
Median time to CAR T-cell infusion in days	29	52	not reported
Received intended ASCT (%) SOC arm	36	32.5	45.6
Cross over per protocol (%) SOC arm	-/-	51	51
Off-protocol CAR T-cell therapy (%) SOC arm	56	-/-	-/-
Safety:			
Grade ≥ 3 TEAEs Experimental arm vs. SOC	91 vs. 83	75 vs. 86	92 vs. 89
CRS: any grade ≥grade 3 in % (Experimental arm)	92	61	48
	6	5	1
ICANS any grade ≥grade 3 in % (Experimental arm)	60	10	7
	21	2	4
Outcome			
ORR/CR in % Experimental arm vs. SOC	83/65 vs. 50/32	46/28 vs. 43/28	87/74 vs. 49/43
Median EFS (month) Experimental arm vs. SOC	10.8 vs. 2.3	3 vs. 3	Not reached vs. 2.4
EFS HR (95% CI)	0.42 (0.33 to 0.55)	1.07 (0.82–1.40)	0.36 (0.243–0.522)
Median PFS (month) Experimental arm vs. SOC	14.7 vs. 3.7	Not reported	Not reached vs. 6.2
PFS HR (95% CI)	0.51 (0.38 to 0.67)	Not reported	0.40 (0.261–0.615)
Median OS (month) Experimental arm vs. SOC	Not reached vs. 31.1	16.0 vs. 15.3	Not reached vs. 29.9
OS HR (95% CI)	0.73 (0.54 to 0.98)	Not reported	0.72 (0.443–1.183)
Median follow-up (month)	47.2	10	17.5

Abbreviations: EFS: event-free survival; PD: progressive disease; PR: partial remission; CR: complete remission; ORR: overall response rate; SOC: standard of care; TEAE: treatment-emerged adverse event; CRS: cytokine release syndrome; ICANS: immuno-cell-associated neurotoxicity syndrome.

**Table 3:** CIBMTR comparison ASCT vs. CAR T-cell therapy.

Study	Shadman, M. et al., *Blood* 139, 1330–1339 (2022) [40]			Shadman, M. et al., *Blood* 2023; 142 (Supplement 1): 781. [41]		
	ASCT	CAR T	*p*-Value	ASCT	CAR T	*p*-Value
Histology	Relapsed DLBCL/HGBL/PMBL			Relapsed DLBCL or PMBL		
Remission prior cellular therapy	Partial remission (per international working group criteria) Patients with negative PET scan (Deauville 1–3) were excluded			Complete remission		
Year of treatment	2013–2019	2018–2019		2015–2021	2018–2021	
Patient number	266	145		281	79	
Baseline characteristic						
Median age, y (range)	58 (18–80)	60 (24–91)	0.07	59 (NR)	64 (NR)	
Refractory to first-line therapy (%)	160 (60)	79 (55)	0.61	20%	29%	0.22
Early treatment failure (within 12 month)				58%	72%	0.02
Elevated LDH before ASCT/CAR T				31%	37%	0.04
Time from diagnosis to ASCT or CAR T			0.30	NR	NR	
<12 month	103 (39)	64 (44)				
>12 month	162 (61)	81 (56)				
Lines of therapy before ASCT or CAR T	1 2					
Median (range)	2 (1–6)	3 (2–11)	<0.001	3	2	<0.01
More than 2 lines, no. (%)	89 (33)	97 (67)	<0.001	NR	NR	
Imaging before treatment						
PET or PET/CT no. (%)	222 (83)	126 (87)	0.36	99.6%	90%	<0.01
Year of cellular therapy						
2018 and after	66 (20)	145 (100)	<0.001	NR	NR	
Median follow-up, month (range)	38 (3–79)	12 (3–26)		49.7 (3–95)	24.7 (3–39)	
CAR Product						
Axi-cel	-/-	145 (100)		-/-	46%	
Tisa-cel	-/-	0		-/-	53%	
Liso-cel	-/-	0		-/-	1%	
Outcome						
Non-relapse mortality 100 d, % (95% CI)	4 (2–7)	2 (0–5)	0.3	5.9	4.1	0.67
2 y PFS % (95% CI)	52 (46–58)	42 (30–53)	0.1	66.2	47.8	<0.001
2 y OS % (95% CI)	69 (63–74)	47 (33–60)	0.004	78.9	65.6	0.037
2y Progression/relapse % (95% CI)	40% (33–46)	52 (41–63)	0.05	27.8	48	<0.001

Abbreviations: NR: not reported; 95% CI: 95% confidence interval; PFS: progression-free survival; OS: overall survival.

## Data Availability

No new data were created or analyzed in this study.
